# Audit of obstetric critical care admissions in a high-risk population

**DOI:** 10.1186/cc12447

**Published:** 2013-03-19

**Authors:** K El-Boghdadly, J Aron, DN Onwochei

**Affiliations:** 1 Princess Royal University Hospital, London, UK; 2 University Hospital Lewisham, London, UK

## Introduction

South-east London (SEL) presents unique challenges to healthcare providers due to its diverse demographic. The high levels of poverty, immigration and psychiatric illness impact delivery of obstetric care. These were identified as risk factors for poor outcome in the latest CMACE report [1]. The Intensive Care National Audit and Research Centre (ICNARC) produced data on obstetric critical care admissions in 2007 [2]. We reviewed the obstetric critical care admissions in three SEL hospitals and compared this with the national average determined in the ICNARC and CMACE data.

## Methods

All critical care admissions in three high-risk obstetric units in SEL (1 August 2009 to 31 July 2011) were screened for patients who were currently or recently pregnant. We compared local results with national data by ICNARC and CMACE.

## Results

**Figure 1:**
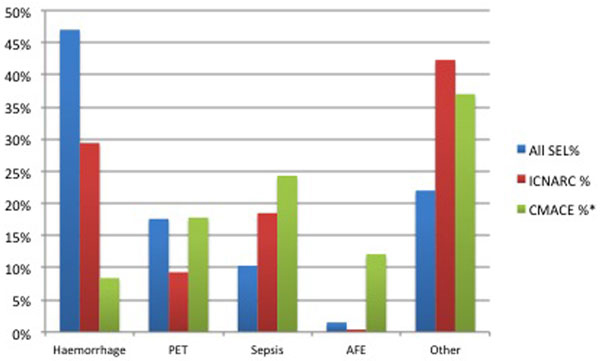
**Causes for critical care admission in SEL, CMACE and ICNARC**.

There were 68 obstetric critical care admissions in the SEL hospitals within the audited time frame. The mean age was 30.05 in ICNARC data compared with 33.93 in SEL. Average APACHE II scores were lower in SEL compared with the ICNARC data, but length of stay was greater in SEL (2.72 days) compared with ICNARC (1.5 days). Haemorrhage was the most common reason for admission in SEL, whilst sepsis was the leading cause of death according to the latest CMACE report (Figure 1).

## Conclusion

Data from national audits may guide protocol, but services must be tailored to local circumstances. SEL has unique population characteristics and obstetric critical care admissions differ significantly from national statistics; in particular, haemorrhage is over-represented in our region. Critical care services were generally required for a short period of time; during this period, routine postpartum care may be omitted as treatment priorities differ. Dedicated critical care services on the labour ward may be a way to combine postnatal care with transient high-dependency requirements. This may enhance patient experience and prove cost-effective.
